# Ulnar dimelia – a review of 24 cases

**DOI:** 10.1177/17531934231196418

**Published:** 2023-09-08

**Authors:** Mona I. Winge, Stéphane Guéro, Vladimir Zavarukhin, Pasi Paavilainen, Carla Baldrighi, Anders Kjørup, Wiebke Hülsemann

**Affiliations:** 1 Division of Orthopaedic Surgery, Oslo University Hospital, Oslo, Norway; 2 Institut de la main, Clinique Bizet, Paris, France; 3 Saint Petersburg State University Hospital, Vasilievsky island, Saint-Petersburg, Russia; 4 Tampere University Hospital, Central Hospital, Tampere, Finland; 5 Al Jalila Children's Specialty Hospital, Dubai, UAE; 6 Rigshospitalet, Copenhagen, Denmark; 7 Children`s Hospital Wilhelmstift, Handsurgery Department, Hamburg, Germany

**Keywords:** Absent radius, congenital upper limb anomaly (CULA), duplicated ulna, polydactyly, true mirror hand, ulnar dimelia

## Abstract

Ulnar dimelia is a very rare unilateral congenital upper limb anomaly (CULA) affecting the whole extremity. Treatment remains difficult because of the complexity and multi-level involvement. Twenty-four cases with duplicated ulna, absent radius and polydactyly from seven European centres were reviewed according to a structured list of parameters. At first consultation, median age 8 months (1–178), the shoulder movement was good in 17 patients or poor in six, and the median passive elbow range of motion was 20° (0°–90°). The resting wrist position was flexed in 22/24 patients. Following stretching and splinting, elbow surgery included resection of the lateral proximal ulna in 11 patients and muscle transfers in six to improve passive movement and increase active elbow motion, respectively. Tendon transfers were performed in eight wrists and a pollicization or pseudo-pollicization in 23 patients. Overall, patients demonstrate acceptable function postoperatively. Guidelines for treatment of this severe CULA are presented.

**Level of evidence:** IV

## Introduction

Ulnar dimelia is an extremely rare congenital upper limb anomaly (CULA), first described by Rueff in 1587. Most reports in the literature are single cases. A recent case series of 13 patients includes seven with a true ulnar dimelia (Kumar et al., 2022). Due to its rarity, cadaveric studies have been helpful in providing important insights on the anatomical variations (Askari et al., 2016; Barton et al., 1986a).

Although the term ‘mirror hand’ is often used in this condition, ulnar dimelia affects the whole extremity including the shoulder girdle (Barton et al., 1986b;  Davis and Farmer, 1958; Harpf and Hussl, 1999; Schmit et al., 2000). It is typically characterized by an absent radius, which is instead replaced by a duplication of the ulna and polydactyly of the fingers with an absent thumb. The condition is classified as IA2iii and IB2v malformations/abnormal axis formation of entire upper limb and hand plate anteroposterior axis using the Oberg–Manske–Tonkin (OMT) classification and mirror hand Type IA and IB according to Al-Qattan`s classification (Al-Qattan et al., 1998; Goldfarb et al., 2020).

The cause of ulnar dimelia is thought to be due to the duplication of the zone of polarizing activity (ZPA), normally located on the posterior part of the developing limb bud. This area produces a morphogen called Sonic hedgehog (Shh), which acts on the cells in the area as they develop and guides anterior–posterior growth and identity. When the ZPA is duplicated, resulting in ectopic Shh deposited on the anterior limb bud as well, both sides of the developing forearm and hand develop as a mirror image with characteristic ulnar features, namely an ulna instead of the radius (ulnar dimelia) and fingers instead of the thumb (Riddle et al., 1993). Lam and coworkers have experimentally demonstrated how preaxial polydactyly, radial aplasia and ulnar dimelia are part of a single disease spectrum likely involving ectopic Shh activities (Lam et al., 2019). Despite these advances in knowledge about the aetiology, there has been no large clinical experience or consensus on treatment options.

The main aim of our study is to assess, comprehensively, a large number of ulnar dimelia cases, based on a pre-determined set of parameters from different centres across Europe.

## Methods

**Figure 1.**
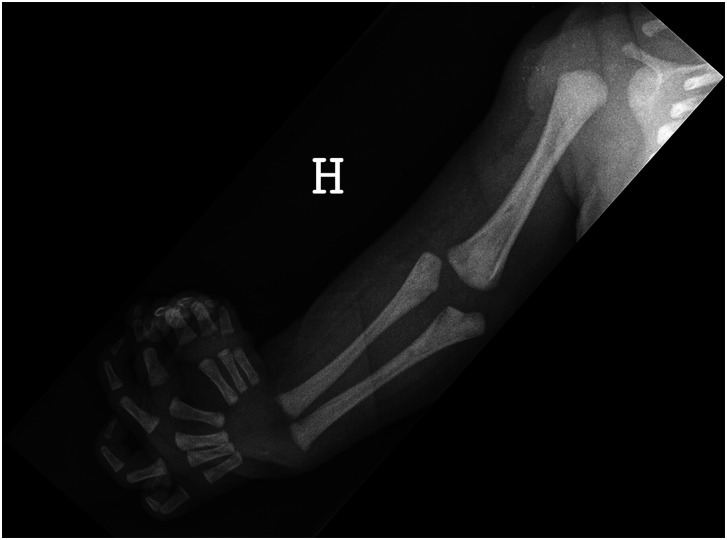
Radiograph of Case 23 shows a normal gleno-humeral joint, a dysplastic broad distal humerus, an absent radius, duplicated ulna and polydactyly.

We included ulnar dimelia cases (absent radius, duplicated ulna and polydactyly) who presented consecutively at our respective clinics from 1981 to 2022 (Figure 1). Twenty-four patients from seven European centres (Oslo, Paris, Saint Petersburg, Tampere, Florence, Copenhagen, Hamburg) were retrospectively reviewed. The study conforms to ethical norms and standards in the declaration of Helsinki and the national ethical guidelines of the seven European centres.

In March 2020, the participants at the Arctic CULA symposium on Svalbard, Norway, were invited to include their ulnar dimelia cases. A list of parameters was created by author MIW and reviewed by co-authors WH and VZ. The list was sent to participants for completion.

Parameters included general patient medical history, radiological assessment, preoperative status of the upper extremity and range of motion (ROM) at first consultation, non-surgical and surgical treatment, postoperative ROM and functional testing at last follow-up. We performed a retrospective review of patient medical records and radiological imaging. All data was collected and analysed by MIW.

The rays of the hand were described as either preaxial or postaxial rays according the metapterygial axis (Woltering et al., 2020).

The surgical treatment of the polydactylous hand was either a true pollicization (Buck-Gramcko, 1964) or a pseudo-pollicization. The former refers to the transfer of a digit with its vascular-nerve bundle proximally to its own metacarpal base. The latter comprises the identification of the vascular-nerve bundle and tendons, a proximal metacarpal osteotomy on the first preaxial ray keeping tendinous and muscular attachments, the removal of excess rays, a distal metacarpal osteotomy on the ‘best’ preaxial triphalangeal ray and transfer to the base of the first preaxial metacarpal base, fixation with Kirschner (K)-wires or osteosutures and tendon transfers dependent on findings (Upton and Taghinia, 2010).

We calculated patients’ lateral pinch strength percentage according to age, hand and sex (McQuiddy et al., 2015). Data is presented as median and range.

## Results

Twenty-four patients, which included nine girls and 15 boys, presented at first consultation with a median age of 8 months (1–178) and follow-up of 10 years (1–31). The CULA was unilateral in all cases (eight right and 16 left), with only one case of congenital lower limb anomaly (CLLA) described as flexion contracture in both knees. One child had a positive family history for another type of CULA in a distant relative (transverse deficiency) and there was one consanguinity case with otherwise negative family history. The perinatal medical history of four patients included fever and cold; subchorionic haematoma; perinatal haematemesis and a case of cervical dysplasia and torticollis. Eighteen were described healthy at birth and two have unknown perinatal history.

**Figure 2.**
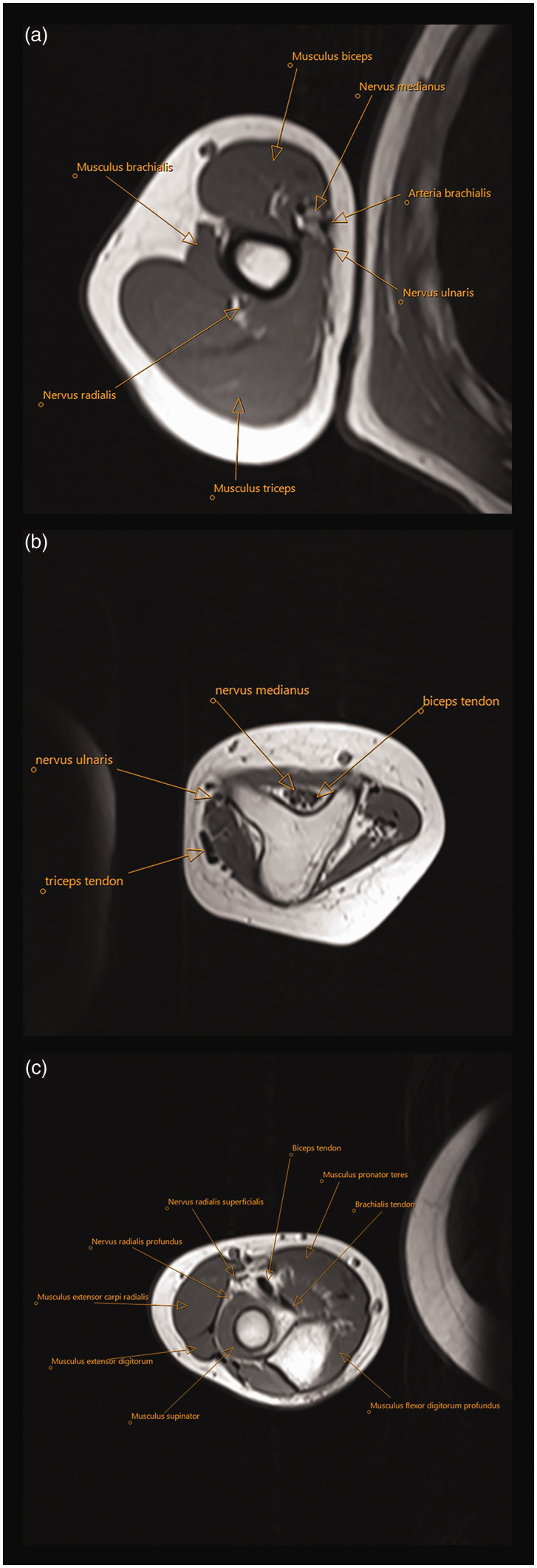
Case 9. MRI sections (a) at mid-upper arm, (b) of the triangular shaped dysplastic distal humerus and (c) just distal to the elbow joint, depicting two proximal ulnae. Identification of anatomical structures of interest such as biceps, brachialis and triceps muscles is possible.

The radiological assessment included radiographs, magnetic resonance imaging (MRI) (*n = *11), ultrasound (US) (*n* = 3), computer tomography (CT) scan (*n* = 9) and CT-angiography (*n* = 2) (Figure 2(a)–(c)). The individual skeletal deformities are detailed in Table S1. Intra-operative doppler (1) confirmed a good arterial signal to all three pre-axial rays.

### Preoperative clinical examination

**Figure 3.**
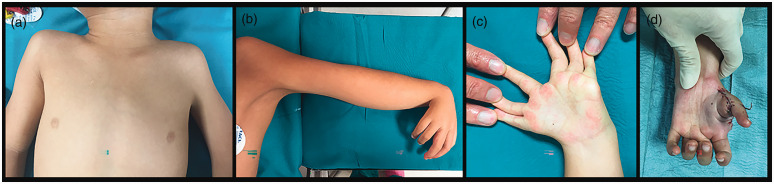
Case 22. (a) Shoulder asymmetry, muscle hypoplasia with slight webbing of the axillary fold left side; (b) absent elbow flexion crease, hyperflexed wrist and polydactylous hand; (c) two natural clefts between preaxial and postaxial rays, and postaxial rays one and two and (d) pollicized hand age 4.9 years.

The preoperative status of the upper extremity at first consultation described the shoulder movement as good in 17 patients and poor in six (Figure 3(a)–(b)) (Table 1). The passive range of motion (PROM) in the elbow was median 20° (0°–90°). A passive elbow ‘abduction’ (passive movement in a lateral direction in the ulno–humeral joint) of 20° and 30° was noted in two elbows. The resting position of the wrist was flexed in 22 cases and neutral in two cases. The median PROM in the wrist was 80° (20°–120°) in 20 patients with absent active motion in five patients and presence of motion in 14. The median active range of motion (AROM) was 50° (20°–80°) in ten patients.

**Table 1.** Preoperative status of the upper extremity at first consultation.

Case	Preoperative status upper extremity										
	Shoulder			Elbow	Wrist			Hand			
	Mobility Good/bad	Active flexion Normal (N)/bad (°)	Active abduction Normal (N)/bad (°)	PROM (°)	Resting position Flexed/neutral/extended (°)	PROM Extension-neutral-flexion (°)	AROM (°)	Triphalangeal rays (*n*)	Biphalangeal rays (*n*)	Pre-axial rays (*n*)	Broader web
1	Good	NA	Bad	0–20 (20)	Neutral	10-0-90 (100)	–10–80 (70)	7	0	3 ^c^	Normal
2	NA	NA	NA	0	Flexed 45 + RD 30	NA	NA	7	0	3	A + C
3	Bad	40	90	0–20 (20)	Flexed 50	20-0-70 (90)	–30–60 (30)	6	0	2	normal
4	Good	N	N	0–60 (60)	Flexed 60	0-0-60 (60)	NA	8	0	4	B
5	Good	N	N	0–40 (40)	Flexed 80	0-0-80 (80)	No	8	0	4	B ^g^
6	Good	N	N	−10–60 (50)	Flexed 30	0-0-60 (60)	–30–50 (20)	7	0	3	NA
7	Good	N	N	0–10 (10)	Flexed 60	20-0-80 (100)	–20–80 (60)	7	0	3	A + B
8	Bad	NA	NA	20–60 (40)	Hyperflexed	NA	NA	7	0	3	NA
9	Good	NA	NA	0–30 (30)	Flexed 40	20-0-80 (100)	0–40 (40)	6	1	3 ^b^	A + C
10	Bad	0	0	0–30 (30)	Flexed 50	40-0-80 (120)	NA	7	0	3 ^b^	NA
11	Good	NA	NA	0–30 (30)	Flexed 50	35-0-80 (115)	0–50 (50)	6	0	2^b^	C
12	Good	N	N	0 (0)	Flexed 75	0-0-75 (75)	No	7	0	3	C
13	Good	N	N	0–10 (10)	Flexed 70	0-0-70 (70)	No	7	0	3 ^b^	C
14	Good	N	N	0–60 (60)	Flexed 30	20-0-100 (120)	10–90 (80)	7	0	4 ^d^	NA
15	Good	N	N	0–10 (10)	Flexed 120	–40–135 (95)	No	7	0	3	B + C
16	Bad ^a^	20	180	10 (0)	Flexed	0-0-30 (30)	No	7	0	3	B
17	Good	170	180	0 (0)	Neutral	10-0-70 (80)	0-0-70 (70)	6	1	3	B
18	Bad	20	20	0–20 (20)	Flexed 80	–70–90 (20)	20	6	0	2	A
19	Good	170	180	0 (0)	Flexed 20	0-0-80 (80)	80	8	0	4	B
20	Good	Good	Good	NA	Hyperflexed	NA	NA	7	1	4 ^e^	B ^g^
21	Good	NA	NA	0	Flexed	NA	NA	6	0	2	NA
22	Bad	10	70	0–90 (90)	Hyperflexed + RD	10-0-90 (100)	Yes	6	0	2	B + C
23	Good	N	N	20–40 (20)	Flexed 20	20-0-20 (40)	Yes	7	0	3	B
24	Good	N	N	0–45 (45)	Flexed 20	10-0-60 (70)	Yes	5	0	2 ^f^	B

A broader web was present between preaxial rays two and three (A); pre- and postaxial rays (B); postaxial rays one and two (C).

a Shoulder fixed at 100° abduction at birth. Treatment with shoulder splint in adduction for 4 months with no effect.

b First preaxial ray is hypoplastic.

c Third preaxial ray is hypoplastic.

d Four preaxial metacarpals of which a rudimentary first metacarpal and three triphalangeal preaxial rays.

e Fourth preaxial ray is hypoplastic.

f Two preaxial metacarpals and one triphalangeal preaxial ray with signs of good hand function without need for grip reconstruction.

g Syndactyly between first and second preaxial rays.

NA: not available; PROM: passive range of motion; AROM: active range of motion; RD: radial deviation.

**Figure 4.**
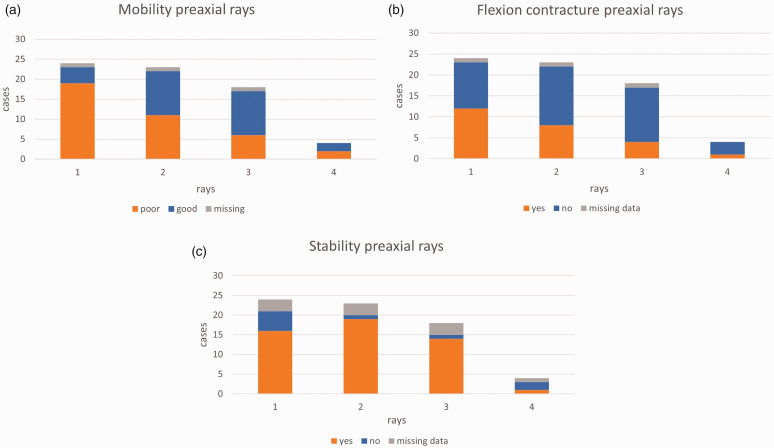
(a) Mobility, (b) flexion contracture and (c) stability in preaxial rays one to four of all 24 cases, indicating the more limited function of preaxial ray one.

The number of preaxial rays were two (*n* = 6), three (*n* = 13) and four (*n* = 5). Three patients had a biphalangeal ray. The number of triphalangeal rays were five (*n* = 1), six (*n* = 7), seven (*n* = 13) and eight (*n* = 3). The preoperative status related to mobility, flexion contracture and stability of the preaxial rays are as shown in Figure 4(a)–(c). The function of the postaxial rays was good (*n* = 20) and poor (*n* = 4). We observed a natural broader web or cleft between the fingers. These were located between preaxial rays two and three (*n* = 4), the pre- and postaxial rays (*n* = 11) and postaxial rays one and two (*n* = 7), of which five children presented two broad webs in their hands (Figure 3(c)).

Six patients presented with a fistula on the forearm of which one was discharging, with a bony spike at the entrance to the fistula. Biopsies confirmed the presence of heterotopic pulmonary tissue in all six patients and *Streptococcus pneumoniae* was isolated from one (Habenicht et al., 2023) (Figure S1). Three patients had dimples on the dorsum of their hands without any fistula (Figure S2).

The shoulder of Patient 16 with a gleno-humeral joint aplasia, fixed at birth at 100° abduction was treated with a splint (Table S1). The treatment was not effective. No shoulder surgery was done in any of our patients.

### Postoperative clinical examination

The treatment of the elbow started with stretching (*n* = 12) and splinting (*n* = 4) and continued secondarily with surgery in 14 patients (Table S2). The median age at surgery was 2.2 years (0.5–15). Initial elbow surgery consisted mainly of a proximal lateral ulna resection (PLUR) (*n* = 11) combined with an anterior humeral condylectomy (*n* = 2), a triceps tendon plasty (*n* = 1), a triceps transfer (*n* = 1) and a brachialis transfer (*n* = 1). Three patients were operated with primary brachialis transfer, distal humeral osteotomy (Patient 16) and resection of an ossified biceps (Figure S3). Secondary surgery included a resection (*n* = 1) or a re-resection (*n* = 2) of the proximal lateral ulna (PLU) combined with arthrolysis (*n* = 1) or a triceps transfer (*n* = 1), a brachialis transfer (*n* = 1) and a latissimus dorsi transfer (*n* = 1). Six muscle transfers were done to increase active elbow motion.

The wrist was initially treated non-operatively with stretching (*n* = 11) and splinting (*n* = 10). Eleven patients received operative treatment at median age 2.5 years (1–16.2), including a wrist distraction (*n* = 2), pseudo-centralization (*n* = 1) or a tendon transfer for wrist extension (*n* = 8) combined with one intercarpal resection and one pollicization. Secondary surgery included tendon transfers at wrist level in two patients, and one with wrist distraction, two with opponensplasties, seven who underwent procedures on the hand and one with distraction-lengthening of forearm and of the humerus.

**Figure 5.**
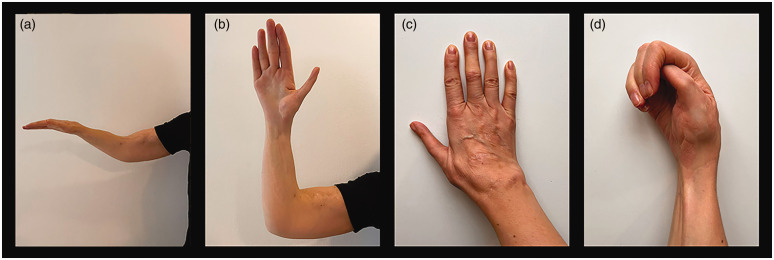
Upper extremity of Case 21: (a) 30 years after latissimus dorsi transfer, extended elbow and wrist in neutral position; (b) flexed elbow and hand 31.5 years after pollicization; (c) dorsal aspect of hand and (d) closed fist.

**Figure 6.**
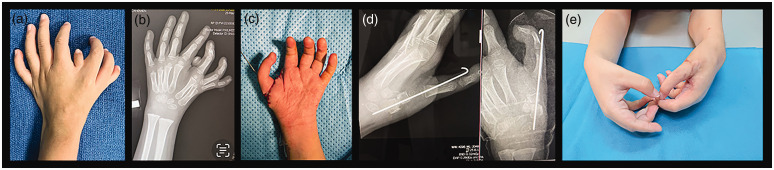
Case 20. (a) Polydactylous hand; (b) radiograph shows seven triphalangeal rays and one biphalangeal ray; (c) after transfer of ‘best’ digit to most preaxial metacarpal base, age 3 years; (d) postoperative radiographs and (e) both hands at age 4.2 years.

Grip reconstruction (*n* = 23) was done using the pre-axial ray one (*n* = 1), two (*n* = 8), three (*n* = 12), four (*n* = 1) and post-axial ray one (*n* = 1) at median age 1.5 years (1–15). One patient with two preaxial metacarpals and one triphalangeal ray is showing signs of good hand function and will be considered for surgery at a later date. The surgical techniques used were either a pollicization (*n* = 17) or a pseudo-pollicization (*n* = 6) (Figures 3(d), 5 and 6).

At last follow-up, shoulder instability was present in three, flexion was poor in four and abduction poor in two (Table 2). The PROM and AROM of 21 elbows were median 70° (0°–100°) and 50° (0°–90°), respectively. Forearm passive rotation was 25° (0°–150°) (18). The passive and active wrist extension were median 13° (–25°–40°) (18) and 0° (–60°–40°) (21).

**Table 2.** Postoperative range of motion.

Case	Age at last functional follow-up (years)	Postoperative range of motion							
		Shoulder			Elbow		Forearm	Wrist	
		Active flexion (good-acceptable-bad)	Active abduction (good-acceptable-bad)	Instability shoulder (yes/no)	Active range of motion (AROM) (°)	Passive range of motion (PROM) (°)	Passive total rotation (°)	Active extension (°)	Passive extension (°)
1	11	NA	NA	NA	0 (0)	0–30 (30)	NA	–35 (–35–70)	NA
2	15.5	Good	Good	No	25–65 (40)	10–90 (80)	150 (70–80)	–60 (–60–90)	–15 (–15–90)
3	12	Bad	Acceptable	NA	0 (0)	0–15 (15)	NA	–15 (–15–35)	20 (20–60)
4	15.7	Good	Good	No	0–70 (70)	0–100 (100)	50 (10–40)	0 (0–90)	NA
5	11	NA	NA	NA	0–50 (50)	0–60 (60)	50	30 (30–80)	30 (30–80)
6	17.5	Good	Good	NA	15–50 (35)	10–60 (50)	0	0 (0–55)	0 (0–55)
7	4.8	NA	NA	NA	NA	NA	NA	NA	NA
8	9.4^a^	NA	NA	NA	0–60 (60)^a^	0–70 (70)^a^	0 ^a^	–20 (–20–60)^a^	0 (0–70) ^a^
9	12.5	Good	Good	No	0–80 (80)	0- 90 (90)	10	20 (20–110)	NA
10	^b^	—	—	—	—	—	—	—	—
11	1.8	Good	Good	Yes	0	0–50 (50)	NA	0	35
12	14	Good	Good	No	0–65(65)	0–80 (80)	30	0	0
13	10	Good	Good	Yes^d^	20–85 (65)	0–100 (100)	45	40 (40–80)	40 (40–90)
14	3	Good	Good	No	0–90 (90)	0–100 (100)	90	10 (10–80)	15
15	2.8	Good	Good	No	20–40 (20)	20–50 (30)	30	0 (0–20)	10
16	34	Bad^c^	Bad	No	0 (0)	0 (0)	0	0 (0–20)	0 (0–20)
17	26	Good	Good	No	85–110 (25)	75–110 (35)	10	10 (10–70)	10 (10–70)
18	10	Bad	Bad	Yes	20–65 (45)	70	0	–25^e^	–25^e^
19	4	Good	Good	No	NA	NA	NA	NA	—
20	4.2	Good	Good	No	5–65 (60)	5–70 (65)	0	30 (30–110)	30 (30–110)
21	17	Bad	Good	No	75	90	65	0	40
22	6	Acceptable	Acceptable	No	0–80 (80)	0–90 (90)	0	10	25
23	17	Good	Good	No	20–40 (20)	20–40 (20)	20	0	0
24	2.5	Good	Good	No	10–60 (50)	10–100 (90)	110 (80–30)	0 (0–90)	20 (20–120)

NA: not available.

a Functional follow-up done before last surgical treatment- follow-up of child and last surgery done elsewhere.

b Functional follow-up not performed.

c Shoulder extension 170°.

d Non-painful inferior gleno-humeral subluxation at maximum flexion.

e Flexion contracture wrist at –25° without active extension.

The active wrist extension was median 10° (0°–40°) for 11 non-operated wrists and median –20° (–60°–0°) for seven wrists after tendon transfers. The PROM and AROM of eight non-operated elbows at median age 14 years were median 73° (0°–100°) and 50° (0°–80°), respectively. The median PROM and AROM of six elbows after PLUR alone, at median age 6.2 years, was 50° (15°–100°) and 28° (0°–90°), respectively. Six elbows operated with a muscle transfer, of which five had undergone earlier PLURs, had median PROM 80° (35°–100°) and AROM 50° (25°–75°), at median age 12 years. The passive forearm rotation after PLUR was median 30° (0°–90°) (9) at median 6 years (0.5–23) after surgery.

### Follow-up

The median age at last functional follow-up was 11 years (1.8–34) and the length of postoperative follow-up was 10 years (1–31) (Table S3). The lateral pinch strength between the first and second fingers was median 13% (6–32) of normative data for 11 patients (McQuiddy et al., 2015). Two patients were followed-up with patient-reported outcome measures (PROMs). The information on activity of daily living (ADL) (9) indicates that seven children manage well whereas two struggle much more. Night splinting of wrists was continued during growth in five patients. The forearm length discrepancy for 17 patients, at median age 11 years (2.8–17.5), was 2.5 cm (0–10).

### Complications

Postoperative complications included singles cases of growth arrest after pseudo-centralization, fracture of first metacarpal after thumb lengthening, displaced K-wire after PLUR and fixation with K-wire, the regrowth of the PLU, an early loss of passive mobility after PLUR and reduced extension in three first pre-axial rays with spontaneous radial nerve recovery after 4 months (Winge and Bye, 2013).

## Discussion

We retrospectively reviewed a case series comprising a large number of true ulnar dimelia cases from different centres across Europe. We confirmed the involvement of the entire upper limb in all cases and reiterated the importance of a detailed examination of the whole upper extremity and good medical record keeping during the first consultation and at follow-up (Hiro et al., 2021).

Radiographs of both upper limbs for radiological comparisons during limb growth should be performed, as limb discrepancy is expected. CT scans of the affected arm as well as US and MRI imaging are helpful, again using the unaffected side for comparison. US has the added bonus of not requiring anaesthesia, but is technically difficult and requires an experienced radiologist (Winge and Bye, 2013). An assessment of vascular supply is recommended in any case of arterial anatomical uncertainty (Askari et al., 2016; Schmit et al., 2000). Arthrography and electromyography (EMG) have also been performed (El Hage et al., 2008; Harpf and Hussl, 1999; Takagi et al., 2014).

Ulnar dimelia affects the shoulder girdle variably, from a mild skeletomuscular hypoplasia to a severe deformity (Schmit et al., 2000). Reconstructive surgery is not always necessary or even possible. Good muscle control is important to limit the clinical impact of any instability, which was confirmed in our patients.

Most elbows are stiff in extension at birth. The symmetrically or asymmetrically duplicated proximal ulnae articulate on a dysplastic distal humerus. The distal humerus is tricondylar, with asymmetrically developed divergent condyles. The proximal medial ulna (PMU) more often has a normal shape, whereas the PLU is dysplastic, blocking elbow flexion and rotation. No true proximal or distal radioulnar joints exist. The elbow joint is comprised mainly of cartilage early in life with the ability to remodel after surgery, improving the articular surface. A coordinated initial stretching regime of the upper extremity with occupational therapist (OT) and/or physiotherapist (PT) should be prioritized. The main aims are to gradually increase the hand-to-mouth motion and incorporate the arm in activity (Irani et al., 2007). This first non-operative stage should increase passive elbow motion and can be sufficient in some elbows (Winge and Bye, 2013). Surgery may, however, be necessary as the next step, consisting in the resection of PLU and bony humeral prominences (Gropper, 1983; Tsuyuguchi et al., 1982). In the recalcitrant elbows, arthrolysis, tenotomies and capsulotomies have been done (Irani et al., 2007). Postoperative physiotherapy is essential. In many cases better elbow flexion and forearm rotation have been obtained, confirmed by our study patients. The regrowth of bone at the PLU is a typical occurrence during growth, incrementally limiting forearm rotation (Tsuyuguchi et al., 1982; Winge and Bye, 2013). If this happens, re-resection of the PLU can be done.

As a second stage, muscle transfers can be considered to increase active elbow motion. Tsuyuguchi et al. (1982) sutured the biceps to the lateral flexor carpi ulnaris after a PLUR. Postoperatively, bow string and bone regrowth were observed. Takagi et al. (2014) sutured the biceps to the PLU, whereas Winge (Winge and Bye, 2013) inserted the brachialis tendon to the PMU, thus also changing the direction of the force vectors. Harpf and Hussl (1999) reported on partial pectoralis transfer. Careful preoperative clinical and radiological assessments are essential in the decision making of muscle transfers. However, muscle amplitude can only be assessed intraoperatively. We suggest an early timing, at around 1–2 years of age, when considering transfer of the not previously stimulated brachialis or biceps due to their anomalous distal humeral insertions.

The wrist presents most often in a flexed resting position with very limited wrist extension. The efficient stretching and splinting will increase passive extension, thus permitting the stimulation of hypoplastic extensor muscles (Irani et al., 2007; Winge and Bye, 2013). The continuous night splinting during growth can limit refractory stiffness and potentially further increase ROM (King and Hoyes, 1982). Tendon transfers to create active dorsal extension have had varied success at long-term follow-up, confirmed by our operated group compared with the non-operated group (Barton et al., 1986b;  Kumar et al., 2022). This could be associated with the severity of the cases and limited quality of both flexor and extensor muscles. We advocate early and continuous conservative treatment of the wrist during growth. The clinical similarities of absent wrist extension in both ulnar dimelia and radial longitudinal dysplasia patients have led to similar treatment options, and recent findings from Lam and coworkers suggest a unifying theory of disease pathogenesis (Lam et al., 2019).

An important step when planning grip reconstruction is good knowledge of the preoperative function and vascular supply to the pre- and post-axial rays. Our review of preaxial mobility, flexion contracture and stability indicate that the most pre-axial ray often functions less well. A true pollicization was performed in most cases, with a pseudo-pollicization as the alternative technique (Barton et al., 1986b;  Gropper, 1983). Other techniques, such as shortening metacarpal osteotomies, have also been done (Al-Qattan et al., 2013; King and Hoyes, 1982). Postoperative splinting can improve functional results (Pintilie et al., 1964; Winge and Bye, 2013).

In this study, six patients presented with a forearm fistula containing pulmonary tissues. A recent report proposes that heterotopic respiratory mucosa can be a potential source of Shh and contribute to the aetiology of ulnar dimelia (Habenicht et al., 2023). Further research in this area should yield interesting information about the spectrum of malformations, including ulnar dimelia, preaxial polydactyly and triphalangeal thumb (Potuijt et al., 2019).

We encourage the use of patient-reported outcomes to assess the effect of this condition on health-related quality of life and outcomes of surgical procedures (Takagi et al., 2014). Our patients’ feed-back on ADL indicates how many manage well despite reduced grip and lateral pinch strength. We recommend using reliable and valid instruments for strength measurements and comparisons with normative data (McQuiddy et al., 2015). Long-term follow-up is warranted as recurrence of deformities is expected during growth leading to changes in upper extremity function.

The main weakness of our study is its retrospective case report design and incomplete available data for certain parameters. We are aware of the heterogeneity within our patient group and the difficulties of comparisons between cases. Hopefully certain principles could be drawn from the long-term follow-up of a large cohort of a very rare CULA. Our study has found that the child’s overall function is acceptable despite joint constraints at several levels and this may be because of the unilateral presentation.

## Supplemental Material

sj-pdf-1-jhs-10.1177_17531934231196418 - Supplemental material for Ulnar dimelia – a review of 24 cases Click here for additional data file. Supplemental material, sj-pdf-1-jhs-10.1177_17531934231196418 for Ulnar dimelia – a review of 24 cases by Mona I. Winge, Stéphane Guéro, Vladimir Zavarukhin, Pasi Paavilainen, Carla Baldrighi, Anders Kjørup and Wiebke Hülsemann in Journal of Hand Surgery (European Volume)

sj-pdf-2-jhs-10.1177_17531934231196418 - Supplemental material for Ulnar dimelia – a review of 24 cases Click here for additional data file. Supplemental material, sj-pdf-2-jhs-10.1177_17531934231196418 for Ulnar dimelia – a review of 24 cases by Mona I. Winge, Stéphane Guéro, Vladimir Zavarukhin, Pasi Paavilainen, Carla Baldrighi, Anders Kjørup and Wiebke Hülsemann in Journal of Hand Surgery (European Volume)

sj-pdf-3-jhs-10.1177_17531934231196418 - Supplemental material for Ulnar dimelia – a review of 24 cases Click here for additional data file. Supplemental material, sj-pdf-3-jhs-10.1177_17531934231196418 for Ulnar dimelia – a review of 24 cases by Mona I. Winge, Stéphane Guéro, Vladimir Zavarukhin, Pasi Paavilainen, Carla Baldrighi, Anders Kjørup and Wiebke Hülsemann in Journal of Hand Surgery (European Volume)

sj-pdf-4-jhs-10.1177_17531934231196418 - Supplemental material for Ulnar dimelia – a review of 24 cases Click here for additional data file. Supplemental material, sj-pdf-4-jhs-10.1177_17531934231196418 for Ulnar dimelia – a review of 24 cases by Mona I. Winge, Stéphane Guéro, Vladimir Zavarukhin, Pasi Paavilainen, Carla Baldrighi, Anders Kjørup and Wiebke Hülsemann in Journal of Hand Surgery (European Volume)

sj-pdf-5-jhs-10.1177_17531934231196418 - Supplemental material for Ulnar dimelia – a review of 24 cases Click here for additional data file. Supplemental material, sj-pdf-5-jhs-10.1177_17531934231196418 for Ulnar dimelia – a review of 24 cases by Mona I. Winge, Stéphane Guéro, Vladimir Zavarukhin, Pasi Paavilainen, Carla Baldrighi, Anders Kjørup and Wiebke Hülsemann in Journal of Hand Surgery (European Volume)

sj-pdf-6-jhs-10.1177_17531934231196418 - Supplemental material for Ulnar dimelia – a review of 24 cases Click here for additional data file. Supplemental material, sj-pdf-6-jhs-10.1177_17531934231196418 for Ulnar dimelia – a review of 24 cases by Mona I. Winge, Stéphane Guéro, Vladimir Zavarukhin, Pasi Paavilainen, Carla Baldrighi, Anders Kjørup and Wiebke Hülsemann in Journal of Hand Surgery (European Volume)
