# Supplementary material for: Ulnar dimelia – a review of 24 cases
Source: J Hand Surg Eur Vol. 2023 Sep 8;48(11):1126–35. doi: 10.1177/17531934231196418 (PMC10785563; doi:10.1177/17531934231196418)
Supplement: sj-pdf-1-jhs-10.1177_17531934231196418 - Supplemental material for Ulnar dimelia – a review of 24 cases [file sj-pdf-1-jhs-10.1177_17531934231196418.pdf]

Supplementary Table 3. Post-operative functional follow-up

Case	Age at last functional follow-up (years)	POST-OPERATIVE FUNCTIONAL TESTING				Continued splinting during growth (yes/no)	Forearm length discrepancy (cm)
		Grip strength (bar-kg)	Lateral pinch strength (kg)		Activities of Daily Living (ADL)		
		Absolute	Absolute	Percentage of normative data %			
**1**	11	NA	NA	-	NA	NA	1.5
**2**	15.5	0[a]	1	11	NA	no	1
**3**	12	NA	0.6	9	NA	NA	3
**4**	15.7	0 [a]	0.9	12	NA	no	NA
**5**	11	NA	1	15	NA	no	1
**6**	17.5	NA	0.4	6	NA	no	2.5
**7**	4.8	NA	NA	-	NA	no	NA
**8**	9.4	NA	0.5	10	NA	NA	2
**9**	12.5	0.1[a]	1	15	weak left side. Difficulties in ADL/eating with knife/fork/ buttoning trousers	NA	3

**10**	5.7[c]	[c]	[c]	[c]	NA	NA	0
**11**	1.8	[d]	[d]	[d]	-	yes	-
**12**	14	1.6[b]	1.6	23	drama/theatre<br>No functional problems in ADL. Can reach to top of head with both arms	night splint wrist	4
**13**	10	8[b]	0.8 (1-2 fingers)<br>1.6 (1-4 fingers)	14 (1-2 fingers)	swimming, floorball, climbing, cycling, play station. No problems in ADL	night splint wrist	1
**14**	3	[d]	[d]	-	good function and bilateral grip between fingers 1-2-3. Uses both hands in daily life	no	1
**15**	2.8	[d]	[d]	-	Weak grip. Prefers lateral grip between fingers 2-3. Stiff elbow. Uses both arms in daily life.	no	2
**16**	34	0	0	-	-	no	50[f1]
**17**	26	4[bb]	2.5	[e]	-	no	15[f]
**18**	10	6[bb]	0.7 (1-2 fingers)<br>2.0 (2-3 fingers)	13 (1-2 fingers)	swimming, gymnastics (paralympics in beam, asymmetric bars, floor)	yes	6
**19**	4	[d]	[d]	-	-	no	3.5
**20**	4.2	[d]	[d]	-	-	no	NA
**21**	17	2[bb]	2.5	32	Not limited in daily life. Pain free hand.<br>Gym, trecking, cooking, office work.	no	5
**22**	6	NA	NA	-	plays well using both hands, good grip, not restricted by affected side	yes	2.5

**23**	17	no	no	-	very limited use on keyboard. Compensatory shoulder movement. Can carry things but prefers healthy side. Used as support. Grip and opposition very difficult	no	10
**24**	2.5	[d]	[d]	-	-	night splint elbow	-

Not available (NA), Grip strength measured with Martin[R] Vigorimeter (bar [a]), Grippit[R] Dynamometer (N converted to kg [b]) and Jamar[R] Dynamometer (kg [bb]). Lateral pinch strength measured with mechanical pinch gauge (kg). Normative data according to McQuiddy (6-19 years of age)

[c] No clinical functional follow-up, only review of x-rays, [d] too young, [e] no normative data for this age group, [f] measurements of whole upper limb length ([f1] pseudo-centralisation done at age 3.5 years resulting in growth arrest of both distal ulnae)
