# Supplementary material for: Ulnar dimelia – a review of 24 cases
Source: J Hand Surg Eur Vol. 2023 Sep 8;48(11):1126–35. doi: 10.1177/17531934231196418 (PMC10785563; doi:10.1177/17531934231196418)
Supplement: sj-pdf-2-jhs-10.1177_17531934231196418 - Supplemental material for Ulnar dimelia – a review of 24 cases [file sj-pdf-2-jhs-10.1177_17531934231196418.pdf]

Supplementary Table 1. Pre- and post-operative radiological assessment

Case	RADIOLOGICAL EXAMINATION					
	PRE-OPERATIVE					POST-OPERATIVE
	RADIOGRAPH	CT	ANGIOGRAPHY/ CT-ANGIO	ULTRASOUND	MRI	RADIOGRAPH
**1**	yes	no	no	no	no	NA
**2**	NA	no	no	no	no	Yes
**3**	NA	NA	no	no	No visible biceps	NA
**4**	yes	no	no	no	Ossified distal biceps	Yes
**5**	yes	no	no	no	no	Yes
**6**	2 ulna facing each other. Ossified biceps	no	no	no	triangular shaped dysplastic distal humerus, 3 asymmetrically shaped condyles, 2 ulna facing each other, 120° open angulation. No biceps or brachial m.	Yes
**7**	yes	no	no	no	no	Yes
**8**	NA	no	no	no	no	NA
**9**	2 ulna facing each other, 6 triphalangeal rays and 1 biphalangeal	yes	no	anterior position of PLU	2 proximal ulna articulating on 2 sides of triangular Y-shaped dysplastic distal humerus with asymmetrically developed condyles, 120° open angulation, 2 triceps inserted on 2 olecranons, hypoplastic biceps and brachial m.	Yes
**10**	Yes	no	no	no	no	Yes
**11**	2 symmetrically developed ulna, 6 triphalangeal rays	yes	no	no flexor muscles at elbow	symmetrical humeral condyle deformity, proximal ulna positioned laterally, no flexor tendon at elbow	Yes

**12**	duplicated ulna, absent radius, 7 rays	no	no	no	AHC -poorly developed biceps tendon adherent to distal part of humerus.	new bone formation at radial proximal ulna
**13**	duplicated ulna, absent radius, 7 rays	[a] CT shoulder (age 7): normal humeral head in GHJ with arms along body, anterior subluxation of humeral head at max. flexion. [a] Dynamic elbow CT (age 13): dysplastic triangular shaped distal humerus and 3 condyle shaped bony prominences. PMU articulates with ulnar condyle, PLU lies anteriorly, "facing" medial one, no proper joint. No active rotation	no	normal shoulder region. Tendon-like structure from upper arm musculature over elbow joint.	no	status post-pollicisation. Good development of 4 post-axial digits. Duplicated hamatum and capitatum in distal row with a carpal bone between them. Duplicated triquetrum and lunate in proximal row. Asymmetrically developed proximal ulna
**14**	duplicated ulna, 7 rays, 1 hypoplastic metacarpal	AHC, small fossa olecrani & coronoidea	no	no	no	new bone formation at PLU. Hyperextended thumb

**15**	duplicated ulna, 7 rays, “radial” deviation of hand	AHC, absent fossa olecrani and fossa coronoidea, “radial” deviation of the hand, hypoplastic preaxial carpal bones	no	no	no	status post-pollicisation, 15° “radial” deviation of wrist. After elbow surgery: shortening of preaxial ulna and radial deviation of hand -30°
**16**	no GHJ, vestigial scapula, clavicula present. Short and bowed humerus. Anterior and inferior shoulder dislocation. 2 olecranons facing each other, articulating with enlarged distal humerus. 7 triphalangeal rays. Abnormal carpal bones.	[a] CT shoulder with 3D reconstruction (age 12): shoulder dislocation associated with glenoid hypoplasia. Ribs deformed by humeral head.	2 ulnar arteries and incomplete arterial palmar arches	no	2 triceps inserted on 1 olecranon. Hypoplastic anterior elbow m. (biceps-brachial m). Axis of both trochleae at an angle of 120° opening anteriorly	yes
**17**	normal scapulo-humeral joint. Normal scapula. 2 olecranons “facing” each other, 2 trochleae. 7 triphalangeal rays. Misshaped lunate.	120° angle between proximal ulna	no	no	muscles available (2 triceps inserted to 1 olecranon). Hypoplastic anterior muscles. Angle of 120° between axes of 2 humeral trochleae.	yes
**18**	no GHJ, vestigial scapula and clavicula present. Distal humeral	120° angle between proximal ulna	yes	no	Triceps muscles available	yes

	dysplasia with 2 olecranons facing each other					
**19**	yes	No	no	no	no	yes
**20**	good GHJ. Common Y-shaped base of 1st and 2nd preaxial metacarpals	No	no	no	no	NA
**21**	no	No	no	no	no	yes
**22**	duplicated ulna and 6 triphalangeal digits	No	no	no	no	no
**23**	enlarged incisura scapula, extra humerus anlage, almost normal PMU with dysplastic PLU, equal size joint surface of both ulna at wrist, complete duplicate of carpal bones. 7 triphalangeal fingers	[a] CT distal to mid-humerus (age 17): dysplastic distal humerus with 3 asymmetrically developed condyle-shaped prominences, PMU shaped like olecranon, less developed PLU with signs of proximal dislocation	no	no	2 ulno-humeral joints between 2 proximal ulna and medial and lateral posterior facets of humerus. Atrophic brachial muscle. Biceps and triceps well developed. No visible distal insertion for biceps or brachialis tendons. No brachioradialis. Forearm flexor and extensor muscles present. Thenar and hypothenar muscles present. Normal bone, no synostosis. No annular ligament.	status post- pollicisation, 5 rays
**24**	normal shoulder. Dysplastic broad distal humerus, PMU with normally shaped joint “facing” a dysplastic PLU. Duplicated carpal bones. One triphalangeal preaxial ray with 2 metacarpals	no	no	no	atrophic deltoid and supraspinatus m. Normal rotator cuff. Lateral head of triceps not visible but well developed long and medial of triceps. Biceps muscle visible proximally with fibrous cord inserted on distal humerus and lateral olecranon. Brachial m. not visible.	Status post-resection PLU

Not available (NA), gleno-humeral joint (GHJ), proximal lateral ulna (PLU), proximal medial ulna (PMU), anterior humeral condyle (AHC)

[a] CT examinations done in 3 older patients
