# Supplementary material for: Ulnar dimelia – a review of 24 cases
Source: J Hand Surg Eur Vol. 2023 Sep 8;48(11):1126–35. doi: 10.1177/17531934231196418 (PMC10785563; doi:10.1177/17531934231196418)
Supplement: sj-pdf-3-jhs-10.1177_17531934231196418 - Supplemental material for Ulnar dimelia – a review of 24 cases [file sj-pdf-3-jhs-10.1177_17531934231196418.pdf]

Supplementary Table 2. Operative treatment

Case	Operative treatment					Post-op elbow treatment	Post-op treatment after grip reconstruction			Age surgery (years)		
	Elbow		Wrist		Hand							
	1st	2nd/3rd	1st	2nd/3rd/other	Pollicisation preaxial ray (no)	Post-op immobilisation (weeks)	K-wires (yes/ no)	Cast (weeks)	Splint (yes/ no) (weeks)	Elbow	Wrist/ Other	Pollicisation
**1**	no	no	1) transfer preaxial finger extensor to carpus	2) CO 1st MC	3	-	NA	NA	NA	-	1) 1<br>2) 3.5	1
**2**	no	no	transfer preaxial FCU to carpus + preaxial finger extensor to 2nd MC	no	3	-	no	3	NA	-	1	1
**3**	PLUR+kwire[a]	no	both FCU to carpus	no	1	3	no	no	no	4	6.5	1.2
**4**	no	no	no	1) arthrodesis 1st MCPj<br>2) CO 1st MC	3	-	no	no	no	-	1) 2.8<br>2) 4.5	1.5
**5**	PLUR+kwire[a]	no	no	no	3	4	no	no	no	4	-	1

**6**	1) resection ossified BT	2) PLUR	wrist distraction in external fixator	no	3	no	no	no	no	1) 15.0<br>2) 16.2	16.2	15
**7**	no	no	1) transfer preaxial FCU to carpus	2) CO 1st MC	2	-	no	4	no	-	1) 2.7<br>2) 4.5	2.7
**8**	PLUR	no	1) transfer postaxial FCU to EDC + EPL shortening	2) post-axial FCU to EDC<br>3) wrist distraction<br>4) ECU shortening + transfer FCU to carpus	3	no	yes	NA	NA	10	1) 2.5<br>2) 3.2<br>3) 10<br>4) 10.3	1.5
**9**	no	no	no	no	3	-	no	no	no	-	-	1.3
**10**	no	no	no	no	2	-	no	no	no	-	-	2.2
**11**	PLUR[b]	no	no	no	2 [e1]	5	no	4	wrist/thumb- at night (6)	0.5	-	2.1
**12**	1) PLUR	2) transfer BT to PMU	no	no	2	5	yes	4	yes	1) 0.5<br>2) 3.5	-	1.5
**13**	BT transfer to PMU	no	no	no	3	5	no	4	thumb/ wrist	0,7	-	1.5
**14**	PLUR & AHCR	no	no	CO 1st MC	3	no	no	4	night splint (6)	1.8	2.1	1

**15**	PLUR & AHCR, TT plasty, dorsal capsulotomy	no	1) intercarpal resection + transfer 2 FCU	2) CO 1st MC	[e2]	-	no	4	night splint (6)	2.3	1) 1.7<br>2) 2.7	1
**16**	CO humerus[c]	no	1) pseudo centralization	distraction- lengthening of<br>2) forearm +<br>3) humerus[d]	2 [e3]	-	no	no	no	1	1) 3.5<br>2) 11<br>3) 25	6.5
**17**	1) PLUR & T transfer	2) re-PLUR<br>3) arthrolysis	no	1) Huber transfer<br>2) thumb lengthening- fracture callotasis MC	3	1	no	no	no	1) 2.8<br>2) 4.5<br>3) 10	1) 6<br>2) 14	1.8
**18**	1) PLUR	2) re-PLUR & T transfer	transfer PL to dorsal carpus	no	2 [e4]	no	yes	no	no	1) 4.2<br>2) 5	1.1	1.8
**19**	no	no	no	no	4[e5]	-	yes	no	no	-	1	1
**20**	no	no	no	no	3 [e6]	-	yes	no	no	-	-	3
**21**	1) partial radial PLUR	2) LD transfer + PL graft to PLU	1) transfer pre-axial FCU to ECRB (+ pollicisation)	2) opponensplasty FDS IV	2	5<br>(night splint 1 year)	yes	4	(4)	1) 1<br>2) 3	1) 1.5<br>2) 6.5	1.5

**22**	no	no	wrist distraction in external fixator	no	2	-	yes	6	yes	-	4.1	4.9
**23**	no	no	no	no	3	-	no	4	no	-	-	1
**24**	PLUR + BT transfer to PMU [f]	-	no	no	no	6 [f]	-	-	-	2.1	-	-

Not available (NA), corrective osteotomy (CO), metacarpal (MC), metacarpophalangeal joint (MCPj), proximal lateral ulna (PLU), proximal lateral ulna resection (PLUR), anterior humeral condyle resection (AHCR), lateral epicondyle (LE), proximal medial ulna (PMU), flexor carpi ulnaris (FCU), flexor digitorum superficialis (FDS), extensor pollicis longus (EPL), latissimus dorsi (LD), brachialis tendon (BT), triceps (T), Triceps tendon (TT), passive range of motion (PROM)

[a] Transverse placement of one k-wire through both ulna. [b] no improvement of PROM intra-operatively after PLUR. Gradual improvement in months after surgery. [c] closing wedge corrective osteotomy at distal third of humerus. [d] 2 monorail external fixators on both ulnae (6 cm) and orthofix ring fixator distal humerus (4 cm).

[d] Pseudo-pollicisation (6): [e1] Transfer of 2nd ray to metacarpal base of ray 1; [e2] Transfer of 1st postaxial to base of 1st preaxial metacarpal base and of the 1st preaxial to index position; [e3] Transfer of 2nd ray to base of 1st ray; [e4] Transfer of 2nd ray to base of 1st ray; [e5] Transfer of ray 4; [e6] Transfer of 3rd ray on common Y-shaped base of 1st and 2nd metacarpals.

[f] Contrary to MRI findings, a brachialis muscle was intra-operatively found attached anteriorly on the humerus with corresponding tendon inserted onto the anterior capsule. Good tendon excursion and amplitude. Passive elbow flexion 90°. Very instable lateral proximal ulna. PLUR and BT transfer to PMU done. Post-operative 24-hour treatment in dynamic splint with the elbow positioned at 90° allowing approx. 30° extension.
