# Supplementary figures and images for: Ulnar dimelia – a review of 24 cases
Source: J Hand Surg Eur Vol. 2023 Sep 8;48(11):1126–35. doi: 10.1177/17531934231196418 (PMC10785563; doi:10.1177/17531934231196418)

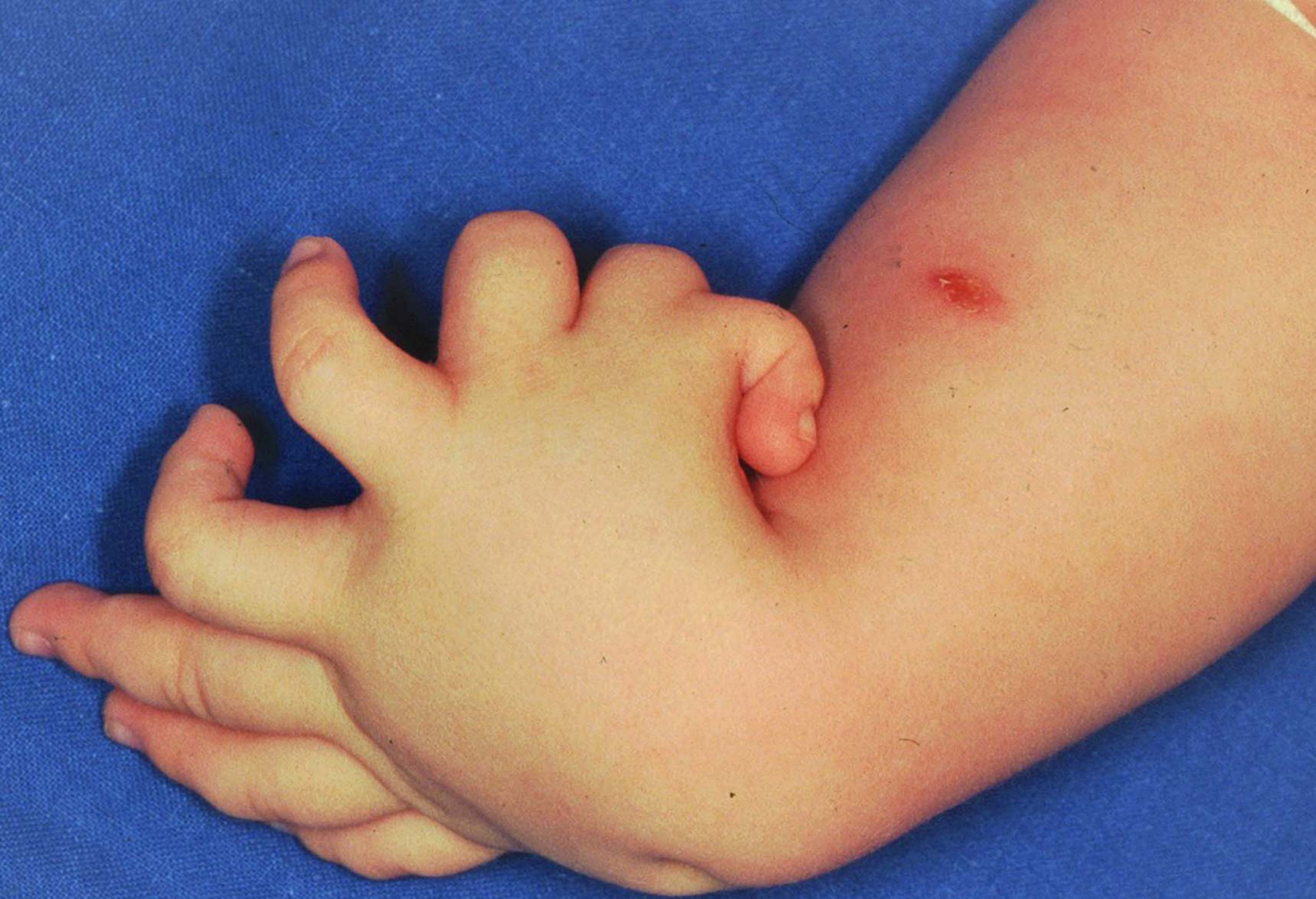

Supplement: sj-pdf-4-jhs-10.1177_17531934231196418 - Supplemental material for Ulnar dimelia – a review of 24 cases [file sj-pdf-4-jhs-10.1177_17531934231196418.pdf]

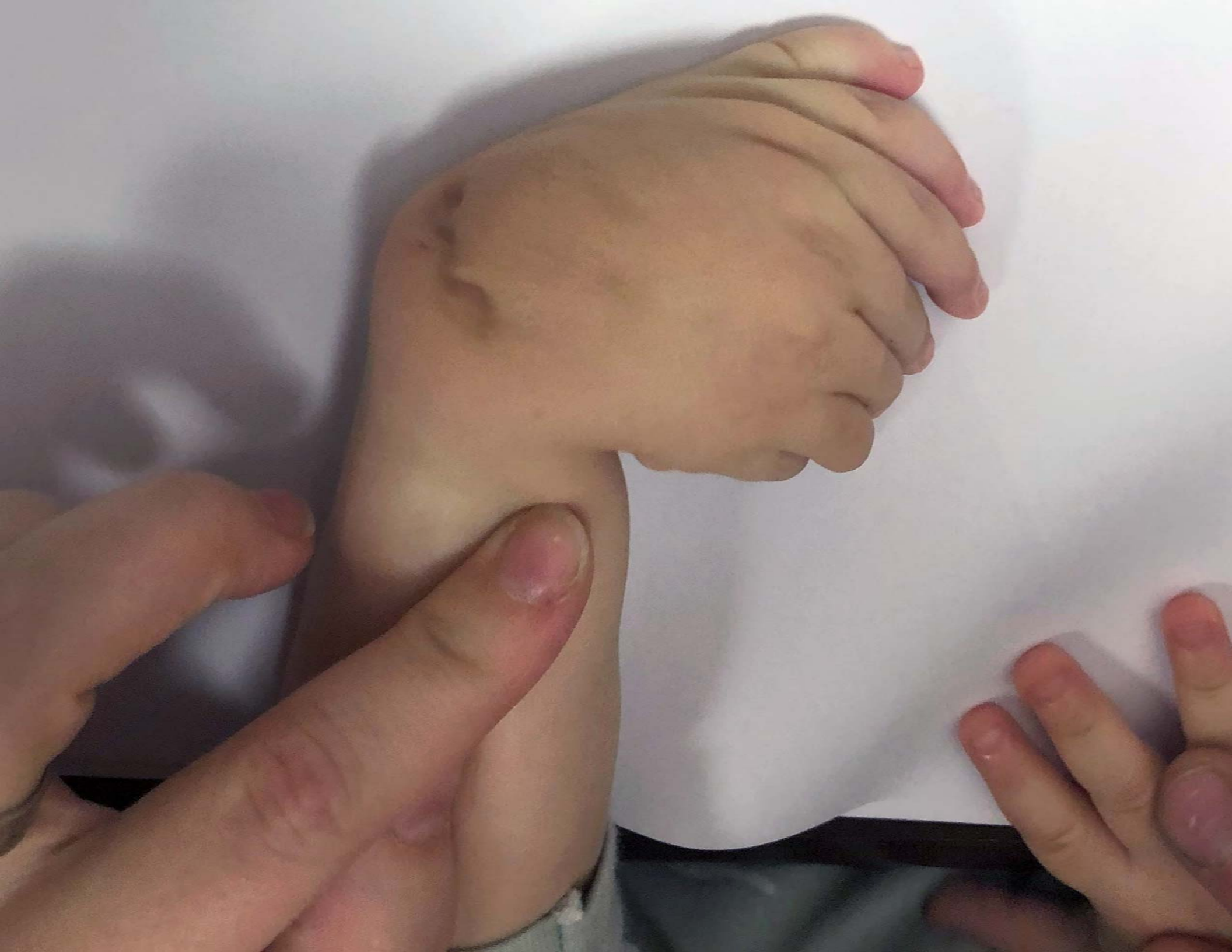

Supplement: sj-pdf-5-jhs-10.1177_17531934231196418 - Supplemental material for Ulnar dimelia – a review of 24 cases [file sj-pdf-5-jhs-10.1177_17531934231196418.pdf]

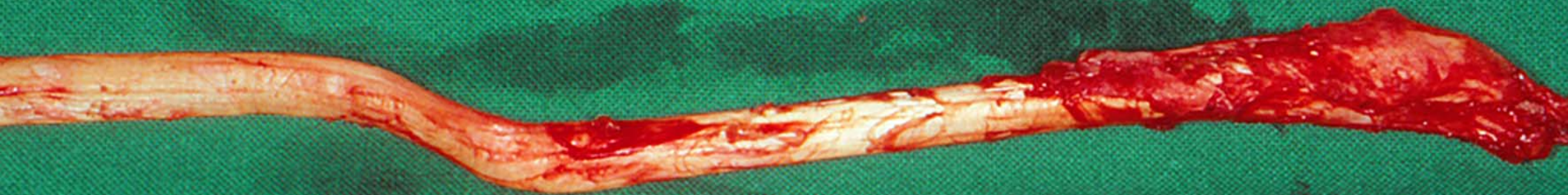

Supplement: sj-pdf-6-jhs-10.1177_17531934231196418 - Supplemental material for Ulnar dimelia – a review of 24 cases [file sj-pdf-6-jhs-10.1177_17531934231196418.pdf]
